# Rectus Sheath Hematoma Triggered by Post-cesarean Anticoagulant Therapy for Intraoperative Acute Pulmonary Thromboembolism: A Case Report

**DOI:** 10.7759/cureus.49034

**Published:** 2023-11-18

**Authors:** Yuki Hirata, Hiroshi Kawamura, Masataka Kato, Yukie Ezaka, Yoshio Yoshida

**Affiliations:** 1 Obstetrics and Gynecology, University of Fukui, Fukui, JPN

**Keywords:** rectus sheath hematoma, twin pregnancy, ovarian vein thrombosis, anticoagulant therapy, case report, pulmonary embolism, cesarean section

## Abstract

Anticoagulant therapy is essential for the prevention or treatment of peripartum venous thromboembolism (VTE). Administration of a therapeutic dose of anticoagulant immediately after cesarean section may result in the formation of a rectus sheath hematoma.

A 32-year-old Japanese woman delivered twin neonates by cesarean section at 37^+5^ weeks of gestation. After the removal of the placenta, the patient suddenly complained of left anterior chest pain and dyspnea with hypotension and desaturation, requiring the administration of oxygen and vasopressors. Postoperative contrast-enhanced computed tomography (CT) revealed pulmonary embolism and massive right ovarian vein thrombosis (OVT). An inferior vena cava filter was placed and continuous intravenous heparin was started. A rectus sheath hematoma was noted on postoperative day 2 (POD 2). On POD 5, heparin administration was temporarily discontinued because of an enlarged rectus sheath hematoma. Approximately 24 hours later, the hemoglobin level recovered, and heparin administration was resumed. No further expansion of the hematoma was observed.

When a rectus sheath hematoma is formed due to treatment with a therapeutic dose of anticoagulant immediately after cesarean section for peripartum VTE, temporary suspension of anticoagulant administration is reasonable to prevent further expansion of the hematoma without fatal complication.

## Introduction

Rectus sheath hematoma is a relatively rare complication following cesarean section [1]. It forms within the rectus sheath due to injury to the superior and inferior epigastric arteries or their branches located between the rectus abdominis muscle and the posterior layer of the rectus sheath or from a tear in the rectus abdominis muscle itself. Risk factors for rectus sheath hematoma include anticoagulation or antiplatelet therapy, subcutaneous abdominal injections, trauma, muscular exertion, surgery, female, pregnancy, and medical comorbidities [2].

Cesarean sections are associated with a potential risk for venous thromboembolism (VTE), reported as 2.6 VTE per 1,000 cesarean sections, and prophylactic anticoagulant therapy is required postoperatively, especially in cases with risk factors for VTE [3,4]. In addition, if perioperative VTE occurs, a therapeutic dose of anticoagulant is required for treatment, which may predispose to the development of an abdominal hematoma. In such cases, delicate postpartum management is needed because anticoagulation therapy increases the risk of perioperative hematoma formation. Herein, we report a rare case of pulmonary thromboembolism developed during cesarean section following massive ovarian vein thrombosis (OVT) in a twin pregnancy. In this case, conservative management was selected for the rectus sheath hematoma induced by postoperative antithrombotic treatment with a therapeutic dose of anticoagulant.

## Case presentation

A 32-year-old Japanese pregnant woman with monochorionic diamniotic twin pregnancy, gravida 3, para 1, without a remarkable medical or family history, was referred to our hospital for perinatal management at 32^+0^ weeks of gestation. Her height was 153 cm, and her body weight was 44.1 kg (pre-pregnancy body mass index was 18.8 kg/m^2^). At 35^+0^ weeks, transabdominal ultrasonography revealed two fetuses with estimated fetal body weights of 2,452 g and 1,772 g, respectively; the discordant rate was 27.7%. Both were cephalic presentations, and the fetal head of the second twin was impacted on the medial aspect of the right anterior superior iliac spine. 

**Figure 1:**
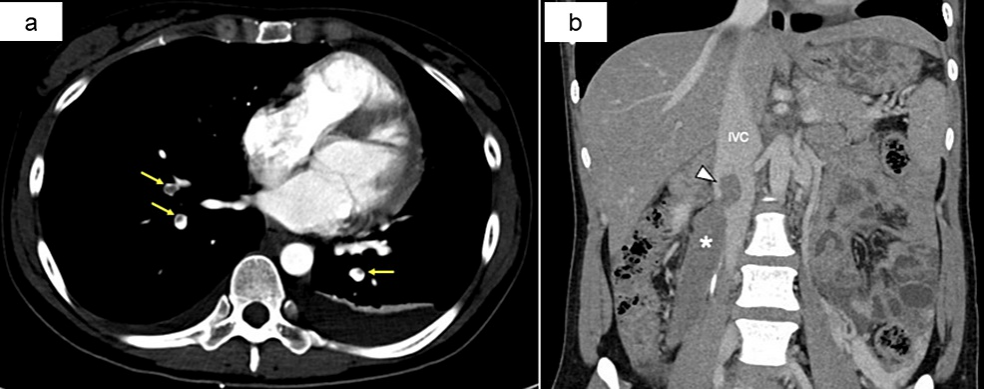
Contrast-enhanced CT just after the cesarean section. (a) Yellow arrows show peripheral pulmonary embolism. (b) The asterisk shows enormous right ovarian vein thrombosis, the size of which was 12×2 cm. The white triangle shows pedunculated thrombi protruding into the IVC. IVC, inferior vena cava; OVT, ovarian vein thrombosis; CT, computed tomography

At 37^+3^ and 37^+4^ weeks, respectively, oxytocin induction of labor was performed, which ultimately failed due to recurrent late decelerations on the cardiotocography of the growth-restricted fetus. Cesarean section was performed at 37^+5^ weeks. Laparotomy was performed by using the modified Joel-Cohen technique under combined spinal-epidural anesthesia. Both neonates were male, with birthweights of 2,900 g and 1,897 g, respectively. Apgar scores were eight and nine and eight and nine at one and five minutes, respectively. Umbilical arterial pH was 7.38 and 7.34 (normal range: 7.15-7.38), respectively. After the removal of the placenta, the patient suddenly complained of left anterior chest pain and dyspnea. Blood pressure dropped from 110/70 mmHg to 67/38 mmHg, and heart rate remained unchanged between 88 and 89 beats per minute. Oxygen saturation was maintained at 99% with nasal prong supplemental oxygen administration (2 L/minute). Hypotension was reversed with vasopressor administration. The uterine contraction was adequate. Although normal blood pressure was maintained, oxygen desaturation to approximately 95% occurred with supplemental oxygen administration via facemask (3 L/minute). Operative blood loss was 1,040 mL including amniotic fluid. Abdominal closure was performed after verification of hemostasis. Subsequently, systemic contrast-enhanced computed tomography (CT) revealed bilateral peripheral pulmonary thromboembolism and a huge right OVT, measuring 12×2 cm with pedunculated thrombi extending into the inferior vena cava (Figure 1a and Figure 1b). There was no deep venous thrombosis of the bilateral lower extremity veins. The immediate postoperative D-dimer level was 213 μg/mL. Intrauterine balloon tamponade was performed in the intensive care for atonic hemorrhage. Five hours later, cardiologists placed an inferior vena cava filter and started continuous intravenous unfractionated heparin (20,000 IU/day) with a target-activated partial thromboplastin time of 1.5-2.5 times the normal range.

**Figure 2:**
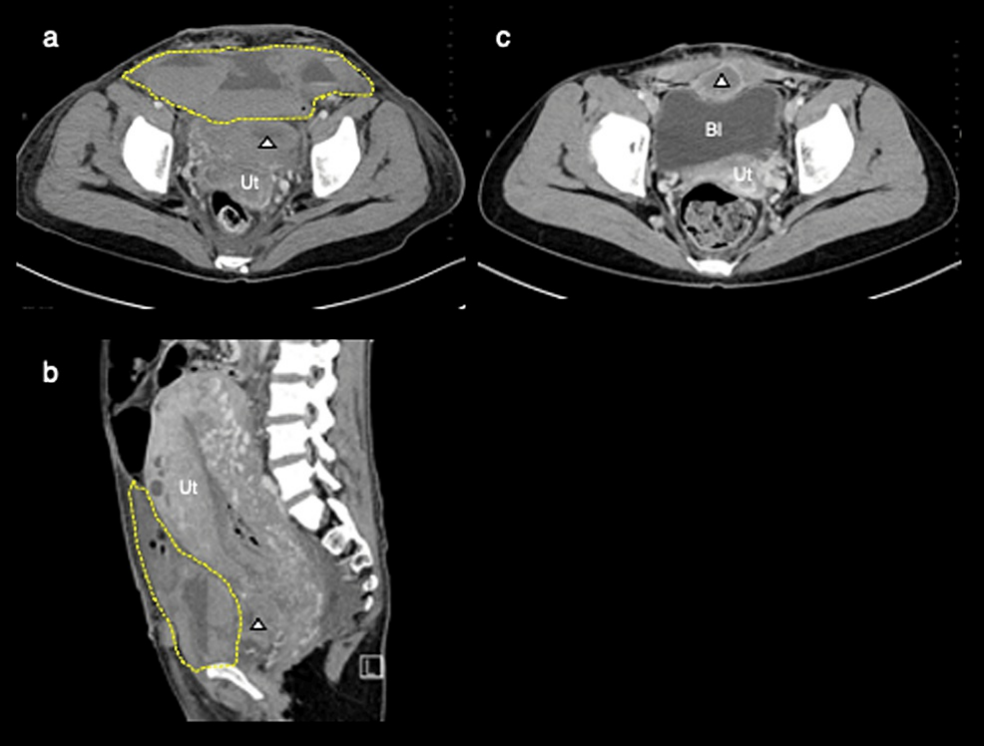
Imaging of rectus sheath hematoma on CT. (a,b) The area surrounded by a yellow dotted line shows the hematoma, including the rectus abdominis muscle, of size 16×5×8 cm on POD 2. The white triangle shows a hematoma at the vesicouterine pouch. (c) On POD 92, the rectus sheath hematoma already disappeared. White triangle shows hematoma at the vesicouterine pouch. Ut, uterus; Bl, bladder; CT, computed tomography; POD, postoperative day

On postoperative day 2 (POD 2), the patient complained of dull lower abdominal pain, and transabdominal ultrasonography revealed a heterogenous echogenic mass, measuring 16×5×8 cm within the abdominal wall. Contrast-enhanced CT demonstrated hematoma formation around or between the bilateral rectus abdominis muscles (Figure 2a and Figure 2b). We consulted with cardiologists and as the patient’s vital signs remained stable, the administration of heparin was continued at a reduced dose (16,000 IU/day); the patient received a total of six units of red blood cells.

On POD 5, the hemoglobin level was 6.5 g/dL, and transabdominal ultrasonography showed that the hematoma had increased in size. As a result, heparin was temporarily stopped and the patient received four more units of red blood cells. Approximately 24 hours later, the hemoglobin level recovered to 9.7 g/dL, and heparin administration was resumed. No further expansion of the hematoma was observed. Antibiotic prophylaxis with cefazolin (3 g/day) was continued for seven days to prevent bacterial infection of the hematoma.

On POD 12, the anticoagulant was changed from intravenous heparin to oral edoxaban after breastfeeding was stopped at the patient's request. The inferior vena cava filter was easily removed on POD 23, and the patient was discharged on POD 28.

Approximately three months later, oral edoxaban was discontinued due to the complete resolution of the right OVT. On POD 92, no rectus sheath hematoma was detected on CT (Figure 2c). Thrombophilia was not detected on her screening blood test on POD 113.

## Discussion

This case shows that huge OVT formed during pregnancy led to intraoperative pulmonary thromboembolism after aortocaval compression was released during cesarean section for twin pregnancies. In addition, the rectus sheath hematoma was induced by postoperative antithrombotic therapy, which posed a clinical dilemma because anticoagulation therapy for VTE resulted in hematoma enlargement. 

Postpartum OVT is a complication in 0.05% of vaginal and 1-2% of cesarean deliveries [5,6]. OVT occurs predominantly on the right side and is thought to result from compression of the right ovarian vein by dextrorotation of the enlarged uterus, increased length of the right ovarian vein, lack of retrograde blood flow, and the presence of multiple incompetent valves [6]. The classic presentation of postpartum OVT includes fever and lower abdominal pain within several weeks, and the differential diagnosis includes other conditions such as endometritis, pyelonephritis, and appendicitis, which are much more important due to the risk of fatal complications like VTE and sepsis [7]. Currently, the significance of detecting asymptomatic peripartum OVT is still unknown [8].

A nationwide Japanese study showed that the risk of VTE is approximately six times higher with cesarean delivery than with vaginal delivery [9]. Rodger et al. previously reported that postpartum pelvic (ovarian or iliac) vein thrombosis, without abdominal pain or fever, was detected by magnetic resonance venography in 30% of vaginal and 46% of cesarean deliveries [10]. Preoperative detection of OVT is desirable, but an effective diagnostic method has not yet been established. In our case, the D-dimer level was significantly elevated at 213 μg/mL when pulmonary thromboembolism was diagnosed. In pregnant women, D-dimer levels increase with gestational age, thus the optimal D-dimer threshold for the prenatal detection of asymptomatic OVT is unknown [11,12]. Kubik-Huch et al. validated the usefulness of ultrasonography with color Doppler, CT, and magnetic resonance imaging (MRI) as imaging modalities for the diagnosis of postpartum OVT. Doppler ultrasound is the least invasive among them but is easily influenced by the patient's body shape or intestinal gas [13]. MRI may be suitable for the evaluation of OVT in pregnant women because it does not require the use of contrast media and the risk of radiation exposure can be avoided. 

This case was a ''near miss'' and could have resulted in maternal death during cesarean section. A recent systematic review showed one maternal death due to pulmonary thromboembolism among 143 cases complicated by postpartum OVT [14]. Salomon et al. reported that the incidence of OVT was 21-fold higher in twin pregnancies than in singleton pregnancies and concluded that cesarean section in twin pregnancies was a significant risk factor for OVT [15]. Therefore, a physician should be cautious about intraoperative and/or postoperative OVT-related pulmonary thromboembolism, especially during and after cesarean section for twin pregnancies.

Fatal complications associated with rectus sheath hematoma rarely occur; however, maternal death due to disseminated intravascular coagulation complicated by rectus sheath hematoma after cesarean section has been reported [16]. Injury to the inferior epigastric arteries may result in clinically significant hematoma formation because they are derived from the external iliac artery, and the area supplied by the inferior epigastric arteries lacks the posterior rectus sheath and is easily propagated by hemorrhage. Sheth et al. demonstrated that 61% of 115 admitted patients who were complicated by rectus sheath hematoma received therapeutic doses of anticoagulants and emphasized that great care should be taken in patients receiving therapeutic doses of anticoagulants [17].

In this case, conservative management was chosen when the rectus sheath hematoma was detected, with close monitoring of maternal vital signs, ultrasound findings of hematoma expansion, and blood tests to ensure no coagulopathy or sepsis. If conservative management of the rectus sheath hematoma had failed, selective arterial embolization would have been considered [18]. In this case, hemostasis was achieved within 24 hours after discontinuation of heparin administration, and there was no rebleeding as maternal blood tests showed no progression of anemia, and her vital signs remained stable. This course of treatment demonstrated that it is reasonable to temporarily suspend antithrombotic therapy, especially after the insertion of an inferior vena cava filter, to prevent further expansion of the rectus sheath hematoma without resulting in fatal VTE-related complications. Thus, looking back at the clinical course, if we had stopped the heparin at the first evidence of hematoma, additional blood transfusions might have been avoided.

In our case, the cesarean section was conducted through the Joel-Cohen technique. There had been many studies about techniques for cesarean section, and the Cochrane review indicated that the Joel-Cohen-based method had advantages compared to Pfannenstiel or conventional techniques in terms of less blood loss, shorter operating time, reduced time for oral intake, less postoperative pain, and fever [19]. However, the risk of perioperative hematoma formation, maternal transfusion, or other serious complications related to techniques of cesarean section has not been fully verified. Regarding the Joel-Cohen technique, the damage of branches of epigastric arteries or a rectus abdominis muscle itself can occur to some extent, especially at the dissection of the rectus sheath. Considering this situation, extreme attention to hemostasis around the rectus sheath muscle is essential before closing the fascia regardless of technique.

## Conclusions

In conclusion, when a cesarean section is performed for a twin pregnancy, initiation of the anticoagulant with a therapeutic dose immediately after surgery should be assumed. Under inferior vena cava filter placement, temporary suspension of therapeutic anticoagulant administration is reasonable to prevent further expansion of rectus sheath hematoma, without any fatal complication related to VTE.
